# Supplementary material for: Anticoagulation trends in adults aged 65 years and over with atrial fibrillation: a cohort study
Source: Open Heart. 2021 Aug 3;8(2):e001737. doi: 10.1136/openhrt-2021-001737 (PMC8336116; doi:10.1136/openhrt-2021-001737)
Supplement: Supplementary data [file openhrt-2021-001737supp001.pdf]

Appendicies

Appendix 1: CPRD codes defining exposure and outcomes

1a: Atrial Fibrillation

CPRD medcode	Readcode	Description
1268	G573200	Paroxysmal atrial fibrillation
1664	G573000	Atrial fibrillation
1757	G573100	Atrial flutter
2212	G573.00	Atrial fibrillation and flutter
23437	G573z00	Atrial fibrillation and flutter NOS
35127	G573300	Non-rheumatic atrial fibrillation
96076	G573500	Persistent atrial fibrillation
96277	G573400	Permanent atrial fibrillation
107472	G573600	Paroxysmal atrial flutter

1b: Anticoagulation

CPRD prodcode	Product Name	Drug substance	Dose	Anticoagulant Type
39119	Rivaroxaban 10mg tablets	Rivaroxaban	10mg	DOAC
39444	Dabigatran etexilate 110mg capsules	Dabigatran etexilate mesilate	110mg	DOAC
39503	Dabigatran etexilate 75mg capsules	Dabigatran etexilate mesilate	75mg	DOAC
39639	Xarelto 10mg tablets (Bayer Plc)	Rivaroxaban	10mg	DOAC
39755	Pradaxa 110mg capsules (Boehringer Ingelheim Ltd)	Dabigatran etexilate mesilate	110mg	DOAC
42474	Pradaxa 75mg capsules (Boehringer Ingelheim Ltd)	Dabigatran etexilate mesilate	75mg	DOAC
46632	Dabigatran etexilate 150mg capsules	Dabigatran etexilate mesilate	150mg	DOAC
46678	Pradaxa 150mg capsules (Boehringer Ingelheim Ltd)	Dabigatran etexilate mesilate	150mg	DOAC
47207	Rivaroxaban 20mg tablets	Rivaroxaban	20mg	DOAC
47353	Rivaroxaban 15mg tablets	Rivaroxaban	15mg	DOAC
47566	Apixaban 2.5mg tablets	Apixaban	2.5mg	DOAC
47925	Xarelto 20mg tablets (Bayer Plc)	Rivaroxaban	20mg	DOAC
48134	Xarelto 15mg tablets (Bayer Plc)	Rivaroxaban	15mg	DOAC

48966	Rivaroxaban 15mg tablets	Rivaroxaban	15mg	DOAC
53740	Eliquis 2.5mg tablets (Bristol-Myers Squibb Pharmaceuticals Ltd)	Apixaban	2.5mg	DOAC
54066	Apixaban 5mg tablets	Apixaban	5mg	DOAC
54451	Rivaroxaban 20mg tablets	Rivaroxaban	20mg	DOAC
56289	Xarelto 20mg tablets (Bayer Plc)	Rivaroxaban	20mg	DOAC
56640	Xarelto 15mg tablets (Bayer Plc)	Rivaroxaban	15mg	DOAC
58594	Eliquis 5mg tablets (Bristol-Myers Squibb Pharmaceuticals Ltd)	Apixaban	5mg	DOAC
62150	Rivaroxaban 2.5mg tablets	Rivaroxaban	2.5mg	DOAC
64500	Xarelto 2.5mg tablets (Bayer Plc)	Rivaroxaban	2.5mg	DOAC
64678	Edoxaban 60mg tablets	Edoxaban tosilate	60mg	DOAC
65247	Edoxaban 30mg tablets	Edoxaban tosilate	30mg	DOAC
65850	Lixiana 60mg tablets (Daiichi Sankyo UK Ltd)	Edoxaban tosilate	60mg	DOAC
65876	Edoxaban 15mg tablets	Edoxaban tosilate	15mg	DOAC
45	Warfarin 1mg tablets	Warfarin sodium	1mg	Warfarin
61	Warfarin 3mg tablets	Warfarin sodium	3mg	Warfarin
833	Warfarin 3mg/5ml oral solution	Warfarin sodium	600microgram/1ml	Warfarin
1781	Warfarin 5mg tablets	Warfarin sodium	5mg	Warfarin
6262	Warfarin 500microgram tablets	Warfarin sodium	500microgram	Warfarin
8466	Marevan 1mg tablets (AMCo)	Warfarin sodium	1mg	Warfarin
8467	Marevan 3mg tablets (AMCo)	Warfarin sodium	3mg	Warfarin
10560	WARFARIN 10 MG TAB			Warfarin
13348	Marevan 5mg tablets (AMCo)	Warfarin sodium	5mg	Warfarin
17965	Marevan 500microgram tablets (AMCo)	Warfarin sodium	500microgram	Warfarin
20754	WARFARIN			Warfarin
23078	Warfarin 1mg Tablet (WB Pharmaceuticals Ltd)	Warfarin sodium	1mg	Warfarin
30202	Warfarin wbp 1mg Tablet (Boehringer Ingelheim Ltd)	Warfarin sodium	1mg	Warfarin
30203	Warfarin wbp 3mg Tablet (Boehringer Ingelheim Ltd)	Warfarin sodium	3mg	Warfarin
31511	Warfarin 3mg Tablet (WB Pharmaceuticals Ltd)	Warfarin sodium	3mg	Warfarin

31937	Warfarin 5mg tablets (Teva UK Ltd)	Warfarin sodium	5mg	Warfarin
33711	Warfarin 5mg Tablet (WB Pharmaceuticals Ltd)	Warfarin sodium	5mg	Warfarin
34019	Warfarin 1mg tablets (IVAX Pharmaceuticals UK Ltd)	Warfarin sodium	1mg	Warfarin
34086	Warfarin 3mg Tablet (Celltech Pharma Europe Ltd)	Warfarin sodium	3mg	Warfarin
34087	Warfarin 1mg Tablet (Celltech Pharma Europe Ltd)	Warfarin sodium	1mg	Warfarin
34088	Warfarin 5mg Tablet (Celltech Pharma Europe Ltd)	Warfarin sodium	5mg	Warfarin
34095	Warfarin wbp 5mg Tablet (Boehringer Ingelheim Ltd)	Warfarin sodium	5mg	Warfarin
34299	Warfarin 1mg tablets (Teva UK Ltd)	Warfarin sodium	1mg	Warfarin
34416	Warfarin 1mg tablets (Kent Pharmaceuticals Ltd)	Warfarin sodium	1mg	Warfarin
34417	Warfarin 3mg tablets (Teva UK Ltd)	Warfarin sodium	3mg	Warfarin
34418	Warfarin 5mg tablets (Mylan)	Warfarin sodium	5mg	Warfarin
34517	Warfarin 1mg tablets (Mylan)	Warfarin sodium	1mg	Warfarin
34526	Warfarin 3mg tablets (Mylan)	Warfarin sodium	3mg	Warfarin
34576	Warfarin 1mg Tablet (Lagap)	Warfarin sodium	1mg	Warfarin
34691	Warfarin 5mg Tablet (Regent Laboratories Ltd)	Warfarin sodium	5mg	Warfarin
34758	Warfarin 3mg tablets (IVAX Pharmaceuticals UK Ltd)	Warfarin sodium	3mg	Warfarin
34864	Warfarin 5mg tablets (IVAX Pharmaceuticals UK Ltd)	Warfarin sodium	5mg	Warfarin
34918	Warfarin 5mg tablets (Actavis UK Ltd)	Warfarin sodium	5mg	Warfarin
36099	Warfarin 1mg/5ml oral suspension	Warfarin sodium	200microgram/1ml	Warfarin
38041	Warfarin sodium 5mg/ml oral suspension	Warfarin Sodium	5mg/5ml	Warfarin
38044	Warfarin 5mg/5ml oral solution	Warfarin sodium	1mg/1ml	Warfarin
39866	Warfarin 1mg tablets (Almus Pharmaceuticals Ltd)	Warfarin sodium	1mg	Warfarin
40143	Warfarin 500microgram tablets (A A H Pharmaceuticals Ltd)	Warfarin sodium	500microgram	Warfarin

43407	Warfarin 3mg tablets (A A H Pharmaceuticals Ltd)	Warfarin sodium	3mg	Warfarin
43408	Warfarin 1mg tablets (A A H Pharmaceuticals Ltd)	Warfarin sodium	1mg	Warfarin
43409	Warfarin 5mg tablets (A A H Pharmaceuticals Ltd)	Warfarin sodium	5mg	Warfarin
43655	Warfarin sodium oral solution	Warfarin Sodium		Warfarin
44866	Warfarin sodium 1mg/ml oral supension SF	Warfarin Sodium	1mg/ml	Warfarin
47944	Warfarin 1mg tablets (Actavis UK Ltd)	Warfarin sodium	1mg	Warfarin
48070	Warfarin sodium tablets	Warfarin Sodium		Warfarin
48869	Warfarin 1mg/ml oral suspension sugar free	Warfarin sodium	1mg/1ml	Warfarin
50000	Warfarin 1mg/ml oral suspension sugar free (A A H Pharmaceuticals Ltd)	Warfarin sodium	1mg/1ml	Warfarin
51484	Warfarin 1mg tablets (Bristol Laboratories Ltd)	Warfarin sodium	1mg	Warfarin
51496	Warfarin 1mg tablets (Phoenix Healthcare Distribution Ltd)	Warfarin sodium	1mg	Warfarin
51509	Warfarin 1mg tablets (APC Pharmaceuticals & Chemicals (Europe) Ltd)	Warfarin sodium	1mg	Warfarin
53745	Warfarin 3mg tablets (Bristol Laboratories Ltd)	Warfarin sodium	3mg	Warfarin
53752	Warfarin 1mg tablets (Alliance Healthcare (Distribution) Ltd)	Warfarin sodium	1mg	Warfarin
54892	Warfarin 1mg/ml oral suspension sugar free (Alliance Healthcare (Distribution) Ltd)	Warfarin sodium	1mg/1ml	Warfarin
54946	Warfarin 3mg tablets (Actavis UK Ltd)	Warfarin sodium	3mg	Warfarin
55316	Warfarin 3mg/5ml oral suspension	Warfarin sodium	600microgram/1ml	Warfarin
56314	Warfarin 3mg tablets (Kent Pharmaceuticals Ltd)	Warfarin sodium	3mg	Warfarin
57032	Warfarin 1mg/ml oral suspension sugar free (Rosemont Pharmaceuticals Ltd)	Warfarin sodium	1mg/1ml	Warfarin

58519	Warfarin 1mg tablets (DE Pharmaceuticals)	Warfarin sodium	1mg	Warfarin
58787	Warfarin 5mg tablets (Alliance Healthcare (Distribution) Ltd)	Warfarin sodium	5mg	Warfarin
58962	Warfarin 3mg tablets (DE Pharmaceuticals)	Warfarin sodium	3mg	Warfarin
59400	Warfarin 500microgram tablets (Sigma Pharmaceuticals Plc)	Warfarin sodium	500microgram	Warfarin
59578	Warfarin 3mg tablets (Phoenix Healthcare Distribution Ltd)	Warfarin sodium	3mg	Warfarin
60589	Warfarin 500microgram tablets (Actavis UK Ltd)	Warfarin sodium	500microgram	Warfarin
60949	Warfarin 5mg/5ml oral suspension	Warfarin sodium	1mg/1ml	Warfarin
62309	Warfarin 500microgram tablets (Kent Pharmaceuticals Ltd)	Warfarin sodium	500microgram	Warfarin
62310	Warfarin 500microgram tablets (AMCo)	Warfarin sodium	500microgram	Warfarin
63071	Warfarin 4mg tablets	Warfarin sodium	4mg	Warfarin
65285	Warfarin 1mg tablets (Crescent Pharma Ltd)	Warfarin sodium	1mg	Warfarin
65496	Warfarin 500microgram tablets (Phoenix Healthcare Distribution Ltd)	Warfarin sodium	500microgram	Warfarin
65746	Warfarin 500microgram tablets (DE Pharmaceuticals)	Warfarin sodium	500microgram	Warfarin
